# Dr. J. Wilson Wishart

**Published:** 1902-08

**Authors:** 


DR. J. WILSON WISHART, of Pittsburg, a graduate of the University of Pennsylvania in 1851, died June 21, 1902, aged 73 years. Dr. Wishart was surgeon of the 140th Pennsylvania Volunteers in the civil war and was one of the chief operating surgeons of the first division, second army corps.

